# A Comprehensive Error Modeling and On-Field Calibration Method for HRG SINS by Tumbling the Hexahedron

**DOI:** 10.3390/s25247645

**Published:** 2025-12-17

**Authors:** Yuanxi Li, Zhennan Wei, Shunqing Ren, Qingshuang Zeng

**Affiliations:** Space Control and Inertial Technology Research Center, Harbin Institute of Technology, Harbin 150080, China; lyx@stu.hit.edu.cn (Y.L.); renshunqing@hit.edu.cn (S.R.);

**Keywords:** strapdown inertial navigation system, hemispherical resonator gyroscope, on-field calibration

## Abstract

**Highlights:**

**What are the main findings?**

- A comprehensive on-field calibration method for HRG SINS is developed, incorporating hexahedral structural errors.
- The proposed 24-position accelerometer calibration and 48-rotation gyro calibration schemes enable simultaneous identification of sensor biases, scale factor errors, installation misalignments, and fixture-induced errors.

**What are the implications of the main findings?**

- The method significantly improves calibration accuracy of both accelerometers and HRGs compared to traditional approaches.
- It relaxes the mechanical precision requirements of the hexahedral fixture, reducing the cost and complexity of on-field calibration.

**Abstract:**

On-field calibration for SINS often uses right hexahedron, but the influence of the structure errors, such as mutual position tolerances towards parallelism or the perpendicularity of two arbitrary planes of the hexahedron, on the calibration accuracy is often neglected. In this paper, a hexahedron structure error model and a comprehensive corresponding SINS calibration error model are developed based on hemispherical resonator gyroscope (HRGs). The proposed method introduces the comprehensive hexahedron errors through defining the normal vectors of the exterior surfaces of the hexahedron. A 24-position calibration scheme is designed to identify accelerometer-related errors, while a 48-rotation scheme is developed to identify gyro-related errors. The complete calibration procedure enables simultaneous identification of hexahedron structure errors, installation misalignments, scale factor errors, and biases. Experimental validation is conducted using a high-precision three-axis turntable, which simulates the hexahedron structure errors. The results show that the proposed method significantly improves the calibration accuracy of both accelerometers and HRGs compared with traditional methods. Furthermore, it reduces the accuracy requirements for the hexahedron structure, thus lowering the cost of SINS on-field calibration.

## 1. Introduction

The strapdown inertial navigation system (SINS) plays a critical role in aerospace, marine, and autonomous navigation applications, where practical navigation accuracy strongly depends on the precise calibration of the inertial measurement unit. The inertial measurement unit typically consists of three accelerometers and three gyroscopes mounted along mutually orthogonal axes. Calibration aims to determine and compensate for the systematic errors of these sensors, such as biases, scale factors, and misalignment errors, thereby ensuring the integrity of navigation performance.

Early research on SINS calibration, such as the work by Bailey at NASA [1], established the theoretical framework of strapdown calibration and alignment using gravity and Earth’s rotation as reference inputs. Later, Nassar [2] improved the INS error modeling approach and emphasized the importance of systematic calibration for accurate navigation. With the development of low-cost and medium-precision MEMS-based IMUs, calibration techniques have evolved rapidly to address the growing needs for accuracy and flexibility in field environments.

Traditional calibration methods generally employ a regular hexahedral fixture to mount the SINS. The cube is sequentially placed in multiple orientations on a horizontal reference slab to generate different known inertial inputs, allowing the estimation of sensor biases, scale factors, and misalignment angles [3,4,5]. Rahimi et al. [6] proposed a multi-position calibration method using rotations to estimate the cross-coupling and scale factor errors of marine-grade IMUs, while Vavilova [7] summarized the calibration problem as a fundamental step in inertial navigation to establish an accurate sensor error model.

However, most conventional methods assume that the hexahedral fixture is geometrically ideal [8,9,10]. In practice, machining tolerances, assembly misalignments, and nonorthogonalities between faces introduce hexahedral structural errors, which lead to deviations in the attitude relationships between the calibration positions. As a result, these unmodeled fixture errors can significantly degrade calibration accuracy, especially for high-precision SINS employing hemispherical resonator gyros (HRGs).

Such unaccounted-for errors can substantially compromise the calibration precision of the SINS error model. In this study, we develop a hexahedral structural error model along with a comprehensive calibration error model for the strapdown inertial navigation systems based on hemispherical resonator gyroscope to field. The proposed approach incorporates the complete set of hexahedral errors by defining the normal vector of each face. We proposed a 24-position calibration protocol for accelerometer error identification, and a 48-rotation procedure to determine gyroscope-related errors. This calibration allows for the simultaneous estimation of hexahedral structural errors, installation misalignments, scale factor deviations, and biases. Experimental verification is carried out on a high-precision three-axis turntable which simulated the hexahedral structural deviations. The results demonstrate that the proposed method substantially enhances the calibration accuracy for both accelerometers and HRGs compared to traditional approaches. Additionally, it relaxes the mechanical precision requirements of the hexahedral fixture, thereby reducing the overall cost of SINS on-field calibration.

Unlike traditional calibration methods which treat the hexahedral fixture as a geometrically ideal rigid body, the proposed method explicitly accounts for the inevitable machining imperfections. In traditional approaches, any deviation in the fixture’s orthogonality or parallelism is incorrectly aliased into the sensor’s biases, scale factors and installation errors, leading to reduced calibration accuracy. In contrast, the method proposed in this paper introduces a normal-vector-based error model to describe the actual geometry of the hexahedron. The distinct advantage of this approach is that it mathematically decouples the fixture-induced structural errors from the intrinsic installation errors of the SINS sensors. Consequently, this method allows for high-precision calibration even when using a fixture with relaxed mechanical tolerances, thereby reducing the manufacturing cost and complexity of the on-field equipment.

## 2. The Calibration System and Establishment of Coordinate Systems

Traditional on-field calibration of Strapdown Inertial Navigation Systems typically employs a regular hexahedral fixture. The gravity-induced specific force and the Earth’s rotation rate are used as two vectors of motion parameters, and the hexahedron is tumbled to provide different excitations for the SINS. However, traditional SINS on-field calibration methods generally neglect the structural errors of the hexahedron. In practice, mutual position errors between the faces of the hexahedron, such as deviations in parallelism and perpendicularity, introduce additional errors during the calibration process, thereby affecting the identification accuracy of the SINS error model.

**Figure 1:**
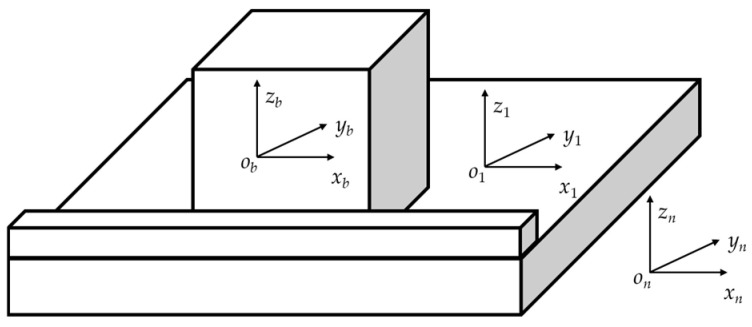
Schematic diagram of the HRG SINS on-field calibration setup.

As shown in Figure 1, the strapdown inertial navigation system based on hemispherical resonator gyroscope is calibrated by placing the hexahedron on the on-field slab. A located block is mounted on the slab to provide positioning. The azimuth of the edge of the located block is precisely determined; therefore, providing precise components of the turn rate of the Earth. The hexahedron is placed tightly against the located block and then rotated on the slab, ensuring that one face of the hexahedron is placed tightly against the located block after each rotation. By changing the orientation of the hexahedron, the input of the SINS is varied. Different attitude transformations are designed to achieve the on-field calibration of the SINS.

To accurately analyze the error propagation mechanism of the strapdown inertial navigation system, several auxiliary coordinate systems are established as follows. The navigation coordinate system *o_n_x_n_y_n_z_n_* is defined such that its three axes point toward the local east, local north, and local up directions, respectively. The slab coordinate system *o*_1_*x*_1_*y*_1_*z*_1_ is defined with its origin *o*_1_ located on the slab. The *o*_1_*z*_1_ axis is parallel to the normal vector of the slab, pointing up and perpendicular to the slab surface. The hexahedron coordinate system *o_b_x_b_y_b_z_b_* is rigidly attached to the strapdown inertial navigation system’s hexahedral structure. These coordinate systems together provide a consistent spatial reference framework that serves as the foundation for subsequent calibration error analysis and the establishment of the system’s comprehensive error model.

## 3. Error Mechanism Analysis

To improve the on-field calibration accuracy of the strapdown inertial navigation system based on hemispherical resonator gyroscope, it is necessary to analyze the error mechanisms involved in the system’s on-field calibration. Based on this analysis, the on-field calibration error model of the system that includes all error sources is established.

### 3.1. Definition of Mutual Position Error of Hexahedron

To improve the accuracy of on-field calibration for strapdown inertial navigation systems based on hemispherical resonator gyroscopes, it is necessary to analyze the effects of the hexahedral structural errors, which are often neglected in traditional calibration methods. Based on this analysis, an accurate error model that incorporates the complete hexahedral calibration errors through normal vectors is established.

The hexahedron coordinate system is defined by the inner normal vector of the first face, the normal vector of the edge shared by the first and second faces, and the projection of the inner normal vector of the second face onto the plane of the first face. It is assumed that the angular deviations between the actual hexahedron and the ideal standard hexahedron are small. 

**Figure 2:**
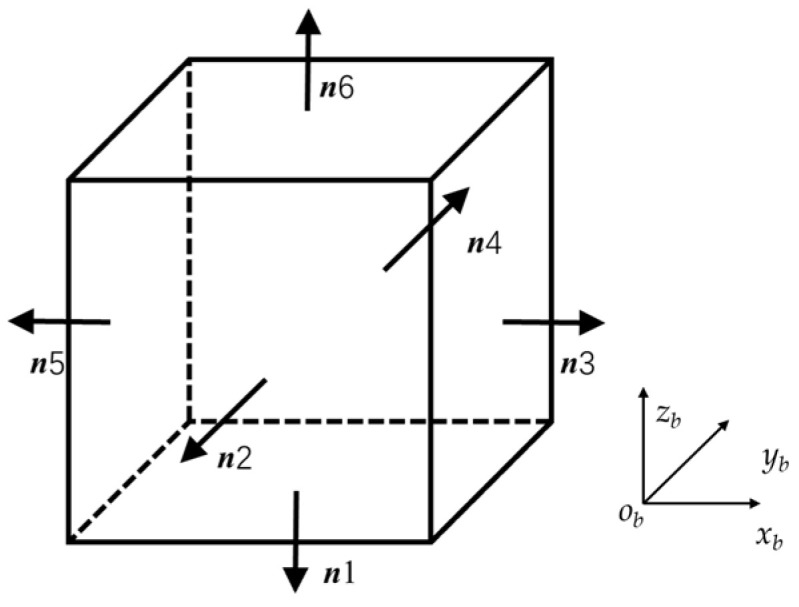
Geometric definition of the hexahedral structure.

The outward normal vector of the first face of the hexahedron is defined as ***n***_1_. The outward normal vector of the second face, ***n***_2_, is determined based on its perpendicularity deviation Δ*θ_x_*_2_ relative to ***n***_1_. Using ***n***_1_ and ***n***_2_ as references, the outward normal vector of the third face, ***n***_3_, is defined, and the remaining faces are defined sequentially in the same manner, which is shown in Figure 2.

Through this definition, nine parameters are employed in this paper to comprehensively represent the parallelism and perpendicularity errors among the faces of the hexahedron, providing a fundamental basis for the subsequent establishment of the SINS calibration error model.

The outward normal vectors of the hexahedron surfaces in the hexahedron coordinate system are defined as shown in Equation (1).

(1) $$\begin{array}{ccc} \begin{array}{l} n_{1}:{\left[\begin{array}{ccc} 0 & 0 & -1 \end{array}\right]}^{T}\\ n_{4}:{\left[\begin{array}{ccc} \Delta \theta_{z4} & 1 & -\Delta \theta_{x4} \end{array}\right]}^{T} \end{array} & \begin{array}{l} n_{2}:{\left[\begin{array}{ccc} 0 & -1 & \Delta \theta_{x2} \end{array}\right]}^{T}\\ n_{5}:{\left[\begin{array}{ccc} -1 & \Delta \theta_{z5} & -\Delta \theta_{y5} \end{array}\right]}^{T} \end{array} & \begin{array}{l} n_{3}:{\left[\begin{array}{ccc} 1 & -\Delta \theta_{z3} & \Delta \theta_{y3} \end{array}\right]}^{T}\\ n_{6}:{\left[\begin{array}{ccc} -\Delta \theta_{y6} & \Delta \theta_{x6} & 1 \end{array}\right]}^{T} \end{array} \end{array}$$

### 3.2. Error Analysis of the Strapdown Inertial Navigation System

Ideally, the three accelerometers and three hemispherical resonator gyroscopes of the strapdown inertial navigation system are perfectly aligned with the three axes of the hexahedron coordinate system.

However, in practice, assembly imperfections introduce installation errors between the sensitive axes of the accelerometers and HRGs and the axes of the hexahedron coordinate system.

To accurately model these deviations, these installation errors are expressed in vector form in this study. The normal vectors of the sensitive axes of the three accelerometers and the three HRGs, represented in the hexahedron coordinate system, are shown in Equation (2).

(2) $$\begin{array}{l} s^{X}={\left[\begin{array}{ccc} 1 & \alpha_{z1} & -\alpha_{y1} \end{array}\right]}^{T}\\ s^{Y}={\left[\begin{array}{ccc} -\beta_{z1} & 1 & \beta_{x1} \end{array}\right]}^{T}\\ s^{Z}={\left[\begin{array}{ccc} \gamma_{y1} & -\gamma_{x1} & 1 \end{array}\right]}^{T} \end{array}\begin{array}{l} g^{X}={\left[\begin{array}{ccc} 1 & \alpha_{z} & -\alpha_{y} \end{array}\right]}^{T}\\ g^{Y}={\left[\begin{array}{ccc} -\beta_{z} & 1 & \beta_{x} \end{array}\right]}^{T}\\ g^{Z}={\left[\begin{array}{ccc} \gamma_{y} & -\gamma_{x} & 1 \end{array}\right]}^{T} \end{array}$$

The three accelerometers and three hemispherical resonator gyroscopes of the strapdown inertial navigation system also exhibit scale factor errors and bias errors. The output error equations of the three accelerometers are shown in Equation (3).

(3) $$\left[\begin{array}{c} N_{x}^{a}\\ N_{y}^{a}\\ N_{z}^{a} \end{array}\right]=\left[\begin{array}{ccc} 1+\Delta K_{ax} & 0 & 0\\ 0 & 1+\Delta K_{ay} & 0\\ 0 & 0 & 1+\Delta K_{az} \end{array}\right]\left[\begin{array}{c} f_{x}^{b}\\ f_{y}^{b}\\ f_{z}^{b} \end{array}\right]+\left[\begin{array}{c} B_{ax}\\ B_{ay}\\ B_{az} \end{array}\right]+\left[\begin{array}{c} n_{ax}\\ n_{ay}\\ n_{az} \end{array}\right]$$

The output error equations of the three HRGs are shown in Equation (4).

(4) $$\left[\begin{array}{c} N_{x}^{g}\\ N_{y}^{g}\\ N_{z}^{g} \end{array}\right]=\left[\begin{array}{ccc} 1+\Delta K_{gx} & 0 & 0\\ 0 & 1+\Delta K_{gy} & 0\\ 0 & 0 & 1+\Delta K_{gz} \end{array}\right]\left[\begin{array}{c} \omega_{ibx}^{b}\\ \omega_{iby}^{b}\\ \omega_{ibz}^{b} \end{array}\right]+\left[\begin{array}{c} B_{gx}\\ B_{gy}\\ B_{gz} \end{array}\right]+\left[\begin{array}{c} n_{gx}\\ n_{gy}\\ n_{gz} \end{array}\right]$$

### 3.3. Error Analysis of the Slab Coordinate System

In the initial position, the slab coordinate system coincides with the navigation coordinate system. However, due to factors such as fixture errors of the slab, there exist leveling errors Δ*θx*_0_ and Δ*θy*_0_, and horizontal zero offset error Δ*γ*_0_ between the slab coordinate system and the navigation coordinate system. The attitude matrix between the navigation coordinate system and the slab coordinate system is given in Equation (5).

(5) $$C_{n}^{1}=\left[\begin{array}{ccc} 1 & \Delta \gamma_{0} & 0\\ -\Delta \gamma_{0} & 1 & 0\\ 0 & 0 & 1 \end{array}\right]\left[\begin{array}{ccc} 1 & 0 & -\Delta \theta_{y0}\\ 0 & 1 & \Delta \theta_{x0}\\ \Delta \theta_{y0} & -\Delta \theta_{x0} & 1 \end{array}\right]$$

### 3.4. The Motion Parameters Inputs of Accelerometer and HRGs

The specific force generated by gravitational acceleration in the navigation coordinate system is $g^{0}={\left[\begin{array}{ccc} 0 & 0 & 1 \end{array}\right]}^{T}$. Then, its representation in the slab coordinate system is given by

(6) $$g^{1}={\left(C_{n}^{1}\right)}^{T}\left[\begin{array}{c} 0\\ 0\\ 1 \end{array}\right]=\left[\begin{array}{c} -\Delta \theta_{y0}\\ \Delta \theta_{x0}\\ 1 \end{array}\right]$$

The Earth’s rotation angular velocity expressed in the navigation coordinate system is ${\left[\begin{array}{ccc} 0 & \omega_{ie}\cos L & \omega_{ie}\sin L \end{array}\right]}^{T}$.

The transformation matrix from the navigation coordinate system to the slab coordinate system is shown in Equation (5). The Earth’s rotation angular velocity expressed in the slab coordinate system is shown in Equation (7).

(7) $$\omega ={\left(C_{n}^{1}\right)}^{T}\left[\begin{array}{c} 0\\ \omega_{ie}\cos L\\ \omega_{ie}\sin L \end{array}\right]=\left[\begin{array}{c} \omega_{ie}\cos L\cdot \Delta \gamma_{0}-\omega_{ie}\sin L\cdot \Delta \theta_{y0}\\ \omega_{ie}\cos L+\omega_{ie}\sin L\cdot \Delta \theta_{x0}\\ \omega_{ie}\sin L-\omega_{ie}\cos L\cdot \Delta \theta_{x0} \end{array}\right]$$

During the on-field calibration of the SINS, the method involves placing two faces of the hexahedron tightly against the slab and the located block, followed by tumbling the hexahedron for calibration. After each tumble, it is ensured that two faces of the hexahedron remain tightly placed against the slab and the located block.

For different calibration positions, traditional on-field calibration of the SINS typically treats it as a rotation around the three axes of the coordinate system to achieve the desired calibration position. However, due to structural errors in the hexahedron, such an equivalent rotation introduces significant errors into the on-field calibration of the SINS.

Traditional on-field calibration methods typically treat the positional changes as ideal rotations around the coordinate axes. Under this assumption, the transformation matrix is simple.

However, this assumption fails when the hexahedral fixture has structural errors. Machining tolerances mean that simply tumbling the fixture does not result in a perfect 90° attitude change relative to the navigation frame. Consequently, treating the motion as an ideal rotation introduces significant errors into the calibration model.

To address this issue, this paper employs a vector-based approach. We define the orientation of the SINS based on the normal vectors of the hexahedron faces. Specifically, when a face is placed tightly against the slab, its outward normal vector is aligned opposite to the slab’s *z*-axis. This geometric constraint allows us to derive the precise input specific force vector regardless of the fixture’s irregularity.

This paper addresses the issue using a vector-based approach. During the on-field calibration of the SINS, the outward normal vector of the face of the hexahedron placed tightly against the slab is opposite to the *z*-axis direction of the slab coordinate system, while the outward normal vector of the face of the hexahedron placed tightly against the located block is opposite to the *y*-axis direction of the slab coordinate system.

Therefore, the unit vectors of the three axes of the slab coordinate system can be expressed in the hexahedron coordinate system. The resulting transformation matrix is given by Equation (8).

(8) $$C_{0}=\left[\begin{array}{c} {\left(v_{B}\times v_{A}\right)}^{T}\\ {\left(v_{B}\times v_{A}\times v_{A}\right)}^{T}\\ {\left((v_{B}\times v_{A})\times (v_{B}\times v_{A}\times v_{A})\right)}^{T} \end{array}\right]$$

where ***v****_A_* is the outward normal vector of the face of the hexahedron placed tightly against the slab, and ***v****_B_* is the outward normal vector of the face of the hexahedron placed tightly against the located block. Normalize the matrix ***C***_0_ to obtain the transformation matrix ***C***_1_.

Taking the position where the first face of the hexahedron is placed tightly against the slab and the fifth face is placed tightly against the located block as an example. The transformation matrix is shown in Equation (9).

(9) $$C_{1}=\left[\begin{array}{ccc} -\Delta \theta_{z5} & -1 & 0\\ 1 & -\Delta \theta_{z5} & 0\\ 0 & 0 & 1 \end{array}\right]$$

The input specific forces of the three accelerometers are shown in Equation (10).

(10) $$\begin{array}{l} \alpha_{x}^{1.5}=\left[\begin{array}{ccc} -\Delta \theta_{y0} & \Delta \theta_{x0} & 1 \end{array}\right]\cdot \left[\begin{array}{ccc} -\Delta \theta_{z5} & -1 & 0\\ 1 & -\Delta \theta_{z5} & 0\\ 0 & 0 & 1 \end{array}\right]\cdot {\left[\begin{array}{ccc} 1 & \alpha_{z1} & -\alpha_{y1} \end{array}\right]}^{T}=\Delta \theta_{x0}-\alpha_{y1}\\ \alpha_{y}^{1.5}=\left[\begin{array}{ccc} -\Delta \theta_{y0} & \Delta \theta_{x0} & 1 \end{array}\right]\cdot \left[\begin{array}{ccc} -\Delta \theta_{z5} & -1 & 0\\ 1 & -\Delta \theta_{z5} & 0\\ 0 & 0 & 1 \end{array}\right]\cdot {\left[\begin{array}{ccc} -\beta_{z1} & 1 & \beta_{x1} \end{array}\right]}^{T}=\Delta \theta_{y0}+\beta_{x1}\\ \alpha_{z}^{1.5}=\left[\begin{array}{ccc} -\Delta \theta_{y0} & \Delta \theta_{x0} & 1 \end{array}\right]\cdot \left[\begin{array}{ccc} -\Delta \theta_{z5} & -1 & 0\\ 1 & -\Delta \theta_{z5} & 0\\ 0 & 0 & 1 \end{array}\right]\cdot {\left[\begin{array}{ccc} \gamma_{y1} & -\gamma_{x1} & 1 \end{array}\right]}^{T}=1 \end{array}$$

Due to the small magnitude of the Earth’s rotation angular velocity, it is insufficient to provide effective inertial vector inputs for the three HRGs of the SINS. In this paper, a method is proposed in which the hexahedron is rotated on the slab to provide inertial vector inputs for the HRGs.

When the first face of the hexahedron is placed tightly against the slab, the projections of the second, third, fourth, and fifth faces onto the slab are shown in Equation (11).

(11) $$\begin{array}{cccc} n_{2}^{\prime }:\left[\begin{array}{ccc} 0 & -1 & 0 \end{array}\right] & n_{3}^{\prime }:\left[\begin{array}{ccc} 1 & -\Delta \theta_{z3} & 0 \end{array}\right] & n_{4}^{\prime }:\left[\begin{array}{ccc} \Delta \theta_{z4} & 1 & 0 \end{array}\right] & n_{5}^{\prime }:\left[\begin{array}{ccc} -1 & \Delta \theta_{z5} & 0 \end{array}\right] \end{array}$$

The tumble angles of the hexahedron can be determined through the projections of the outward normal vectors of the faces onto the slab.

Taking the example where the first face of the hexahedron is placed tightly against the slab, and it tumbles from the second face placed tightly against the located block to the fifth face placed tightly against the located block.

At this point, the angular velocity inputs for the three HRGs of the SINS are composed of the tumble of the hexahedron and the Earth’s rotational angular velocity.

The angular velocity input caused by the tumble of the hexahedron results in the HRG output, which, after integration, is expressed as shown in Equation (12).

(12) $$\begin{array}{l} \alpha_{x}^{1.2.5}=\int \left[\begin{array}{ccc} 0 & 0 & \omega \end{array}\right]\cdot {\left[\begin{array}{ccc} 1 & \alpha_{z} & -\alpha_{y} \end{array}\right]}^{T}dt=\left[\begin{array}{ccc} 0 & 0 & \frac{\pi }{2}+\Delta \theta_{z5} \end{array}\right]\cdot {\left[\begin{array}{ccc} 1 & \alpha_{z} & -\alpha_{y} \end{array}\right]}^{T}=-\alpha_{y}\cdot (\frac{\pi }{2}+\Delta \theta_{z5})\\ \alpha_{y}^{1.2.5}=\int \left[\begin{array}{ccc} 0 & 0 & \omega \end{array}\right]\cdot {\left[\begin{array}{ccc} -\beta_{z} & 1 & \beta_{x} \end{array}\right]}^{T}dt=\left[\begin{array}{ccc} 0 & 0 & \frac{\pi }{2}+\Delta \theta_{z5} \end{array}\right]\cdot {\left[\begin{array}{ccc} -\beta_{z} & 1 & \beta_{x} \end{array}\right]}^{T}=\beta_{x}\cdot (\frac{\pi }{2}+\Delta \theta_{z5})\\ \alpha_{z}^{1.2.5}=\int \left[\begin{array}{ccc} 0 & 0 & \omega \end{array}\right]\cdot {\left[\begin{array}{ccc} \gamma_{y} & -\gamma_{x} & 1 \end{array}\right]}^{T}dt=\left[\begin{array}{ccc} 0 & 0 & \frac{\pi }{2}+\Delta \theta_{z5} \end{array}\right]\cdot {\left[\begin{array}{ccc} \gamma_{y} & -\gamma_{x} & 1 \end{array}\right]}^{T}=\frac{\pi }{2}+\Delta \theta_{z5} \end{array}$$

The Earth’s rotation angular velocity expressed in the slab coordinate system is shown in Equation (7). The angular velocity input caused by the tumble of the hexahedron and the Earth’s rotational angular velocity results in the HRG output, which, after integration, is expressed as shown in Equation (13).

(13) $$\alpha_{z}^{1.2.5}=\frac{\pi }{2}+\Delta \theta_{z5}+\omega_{ie}T\sin L$$

### 3.5. Total Error Model for Calibration

This paper analyzes all error sources in the on-field calibration of the SINS, including slab errors, and hexahedron structural errors, installation errors, scale factor errors, and biases of the three HRGs and three accelerometers. An on-field calibration error model of the SINS was established for different SINS positions.

When the sixth face of the hexahedron is placed tightly against the slab and the fourth face is placed tightly against the located block, the error model of the three accelerometers of the SINS is given by Equation (14).

(14) $$\left[\begin{array}{c} N_{x}^{a}\\ N_{y}^{a}\\ N_{z}^{a} \end{array}\right]=\left[\begin{array}{c} (1+\Delta K_{ax})(-\Delta \theta_{y0}+\Delta \theta_{y6}+\alpha_{y1})+B_{ax}+n_{ax}\\ (1+\Delta K_{ay})(-\Delta \theta_{x0}+\Delta \theta_{x6}+\beta_{x1})+B_{ay}+n_{ay}\\ -1-\Delta K_{az}+B_{az}+n_{az} \end{array}\right]$$

When the second face of the hexahedron is placed tightly against the slab, and the hexahedron tumbles from the third face placed tightly against the located block to the sixth face placed tightly against the located block, the error model of the HRGs is shown in Equation (15).

(15) $$\left[\begin{array}{c} N_{x}^{g}\\ N_{y}^{g}\\ N_{z}^{g} \end{array}\right]=\left[\begin{array}{c} (1+\Delta K_{gx})\alpha_{z}\cdot \frac{\pi }{2}+T\cdot B_{gx}+n_{gx}\\ (1+\Delta K_{gy})(\frac{\pi }{2}-\Delta \theta_{y3}+\Delta \theta_{y6}+\omega_{ie}\sin L\cdot T)+T\cdot B_{gy}+n_{gy}\\ (1+\Delta K_{gz})(\Delta \theta_{x2}-\gamma_{x})\cdot \frac{\pi }{2}+T\cdot B_{gz}+n_{gz} \end{array}\right]$$

## 4. Identification of Error Parameters of SINS

**Figure 3:**
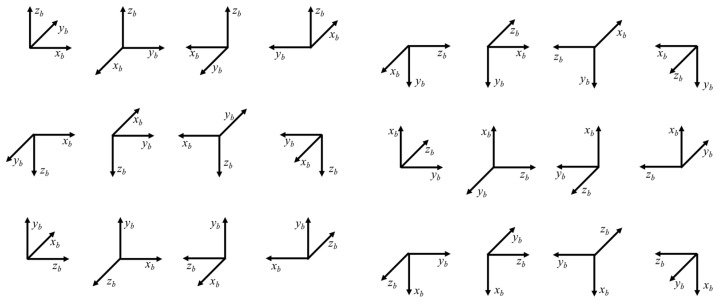
Illustration of the 24-position static calibration scheme.

We designed a 24-position calibration sequence to determine the accelerometer error parameters. The process begins by placing a face of the hexahedron tightly against the slab. While maintaining this contact, the hexahedron is tumbled to align its side faces with the locating block. This is repeated for all six faces, which can be interpreted as orienting the *x*_*b*_, *y*_*b*_, and *z*_*b*_ axes in both upward and downward directions and performing sequential rotations against the positioning plane, which is shown in Figure 3.

First, the first face of the hexahedron is placed tightly against the slab, followed sequentially by placing the second, third, fourth, and fifth faces tightly against the located block. When the second face of the hexahedron is placed tightly against the located block, the specific forces of the three accelerometers in the slab coordinate system are calculated as

(16) $$\begin{array}{l} \alpha_{x}^{1.2}=\left[\begin{array}{ccc} -\Delta \theta_{y0} & \Delta \theta_{x0} & 1 \end{array}\right]\cdot {\left[\begin{array}{ccc} 1 & \alpha_{z1} & -\alpha_{y1} \end{array}\right]}^{T}=-\Delta \theta_{y0}-\alpha_{y1}\\ \alpha_{y}^{1.2}=\left[\begin{array}{ccc} -\Delta \theta_{y0} & \Delta \theta_{x0} & 1 \end{array}\right]\cdot {\left[\begin{array}{ccc} -\beta_{z1} & 1 & \beta_{x1} \end{array}\right]}^{T}=\Delta \theta_{x0}+\beta_{x1}\\ \alpha_{z}^{1.2}=\left[\begin{array}{ccc} -\Delta \theta_{y0} & \Delta \theta_{x0} & 1 \end{array}\right]\cdot {\left[\begin{array}{ccc} \gamma_{y1} & -\gamma_{x1} & 1 \end{array}\right]}^{T}=1 \end{array}$$

When the fourth face of the hexahedron is placed tightly against the located block, the specific forces of the three accelerometers are calculated as

(17) $$\begin{array}{l} \alpha_{x}^{1.4}=\left[\begin{array}{ccc} -\Delta \theta_{y0} & \Delta \theta_{x0} & 1 \end{array}\right]\cdot \left[\begin{array}{ccc} -1 & \Delta \theta_{z4} & 0\\ -\Delta \theta_{z4} & -1 & 0\\ 0 & 0 & 1 \end{array}\right]\cdot {\left[\begin{array}{ccc} 1 & \alpha_{z1} & -\alpha_{y1} \end{array}\right]}^{T}=\Delta \theta_{y0}-\alpha_{y1}\\ \alpha_{y}^{1.4}=\left[\begin{array}{ccc} -\Delta \theta_{y0} & \Delta \theta_{x0} & 1 \end{array}\right]\cdot \left[\begin{array}{ccc} -1 & \Delta \theta_{z4} & 0\\ -\Delta \theta_{z4} & -1 & 0\\ 0 & 0 & 1 \end{array}\right]\cdot {\left[\begin{array}{ccc} -\beta_{z1} & 1 & \beta_{x1} \end{array}\right]}^{T}=-\Delta \theta_{x0}+\beta_{x1}\\ \alpha_{z}^{1.4}=\left[\begin{array}{ccc} -\Delta \theta_{y0} & \Delta \theta_{x0} & 1 \end{array}\right]\cdot \left[\begin{array}{ccc} -1 & \Delta \theta_{z4} & 0\\ -\Delta \theta_{z4} & -1 & 0\\ 0 & 0 & 1 \end{array}\right]\cdot {\left[\begin{array}{ccc} \gamma_{y1} & -\gamma_{x1} & 1 \end{array}\right]}^{T}=1 \end{array}$$

When the third face of the hexahedron is placed tightly against the located block, the specific forces of the three accelerometers are calculated as

(18) $$\begin{array}{l} \alpha_{x}^{1.3}=\left[\begin{array}{ccc} -\Delta \theta_{y0} & \Delta \theta_{x0} & 1 \end{array}\right]\cdot \left[\begin{array}{ccc} \Delta \theta_{z3} & 1 & 0\\ -1 & \Delta \theta_{z3} & 0\\ 0 & 0 & 1 \end{array}\right]\cdot {\left[\begin{array}{ccc} 1 & \alpha_{z1} & -\alpha_{y1} \end{array}\right]}^{T}=-\Delta \theta_{x0}-\alpha_{y1}\\ \alpha_{y}^{1.3}=\left[\begin{array}{ccc} -\Delta \theta_{y0} & \Delta \theta_{x0} & 1 \end{array}\right]\cdot \left[\begin{array}{ccc} \Delta \theta_{z3} & 1 & 0\\ -1 & \Delta \theta_{z3} & 0\\ 0 & 0 & 1 \end{array}\right]\cdot {\left[\begin{array}{ccc} -\beta_{z1} & 1 & \beta_{x1} \end{array}\right]}^{T}=-\Delta \theta_{y0}+\beta_{x1}\\ \alpha_{z}^{1.3}=\left[\begin{array}{ccc} -\Delta \theta_{y0} & \Delta \theta_{x0} & 1 \end{array}\right]\cdot \left[\begin{array}{ccc} \Delta \theta_{z3} & 1 & 0\\ -1 & \Delta \theta_{z3} & 0\\ 0 & 0 & 1 \end{array}\right]\cdot {\left[\begin{array}{ccc} \gamma_{y1} & -\gamma_{x1} & 1 \end{array}\right]}^{T}=1 \end{array}$$

Using the 24-position strapdown inertial navigation system error calibration method designed in this paper, a total of 72 sets of test data were obtained. From these data, we have

(19) $$\left[\begin{array}{c} a_{x}^{1.2}\\ a_{y}^{1.2}\\ a_{z}^{1.2}\\ a_{x}^{1.5}\\ \vdots \\ a_{z}^{5.4}\\ a_{x}^{5.1}\\ a_{y}^{5.1}\\ a_{x}^{5.1} \end{array}\right]=A\cdot \left[\begin{array}{c} 1+\Delta K_{ax}\\ (1+\Delta K_{ax})\Delta \theta_{x0}\\ (1+\Delta K_{ax})\Delta \theta_{y0}\\ (1+\Delta K_{ax})\Delta \theta_{y6}\\ (1+\Delta K_{ax})\Delta \theta_{z4}\\ (1+\Delta K_{ax})\alpha_{y1}\\ (1+\Delta K_{ax})\alpha_{z1}\\ 1+\Delta K_{ay}\\ (1+\Delta K_{ay})\Delta \theta_{x0}\\ (1+\Delta K_{ay})\Delta \theta_{y0}\\ (1+\Delta K_{ay})\Delta \theta_{x6}\\ (1+\Delta K_{ay})\beta_{x1}\\ \left(1+\Delta K_{ay})(\beta_{z1}+\Delta \theta_{z3}\right)\\ \left(1+\Delta K_{ay})(\beta_{z1}+\Delta \theta_{z5}\right)\\ 1+\Delta K_{az}\\ (1+\Delta K_{az})\Delta \theta_{x0}\\ (1+\Delta K_{az})\Delta \theta_{y0}\\ \left(1+\Delta K_{az})(\gamma_{y1}+\Delta \theta_{y3}\right)\\ \left(1+\Delta K_{az})(\gamma_{y1}+\Delta \theta_{y5}\right)\\ \left(1+\Delta K_{az})(\gamma_{x1}+\Delta \theta_{x2}\right)\\ \left(1+\Delta K_{az})(\gamma_{x1}+\Delta \theta_{x4}\right)\\ B_{ax}\\ B_{ay}\\ B_{az} \end{array}\right]+\varepsilon$$

where *A* is a 72 × 24 structure matrix containing only elements 0, 1, and −1, and its rank is 24. Therefore, $\det(A^{T}A)\neq 0$ and the parameters Δ*K_ax_*, Δ*K_ay_*, Δ*K_az_*, *B_ax_*, *B_ay_*, *B_az_*, Δ*θ_x_*_0_, Δ*θ_y_*_0_, Δ*θ_y_*_6_, Δ*θ_z_*_4_, Δ*θ_x_*_6_, *α_y_*_1_, *α_z_*_1_, *β_x_*, *β_z_*_1_ *+* Δ*θ_z_*_3_, *β_z_*_1_ *+* Δ*θ_z_*_5_, *γ_y_*_1_ *+* Δ*θ_y_*_3_, *γ_y_*_1_ *+* Δ*θ_y_*_5_, *γ_x_*_1_ *+* Δ*θ_x_*_2_, *γ_x_*_1_ *+* Δ*θ_x_*_4_ can be identified using the least squares method.

Since the Earth’s rotation rate is too small to provide effective excitations for the three hemispherical resonator gyroscopes of the strapdown inertial navigation system, a set of 48 rotations was designed to serve as the input excitation for the three HRGs.

**Figure 4:**
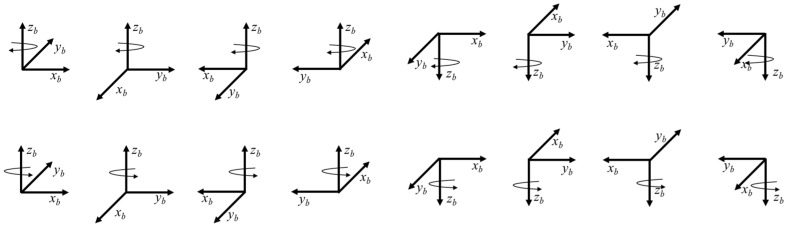
Illustration of the 48-rotation dynamic calibration scheme.

The procedure involves aligning the *x_b_*, *y_b_*, *z_b_* axes of the hexahedral coordinate system upwards and downwards, and then consecutively rotating the hexahedron coordinate system by 90° about the slab’s normal vector. Each face of the hexahedron is placed tightly against the located block. Figure 4 illustrates the rotation procedure when the *z_b_* axis is directed toward the up and down.

By performing the rotations sequentially, the angular input of the HRG aligned with the *z*-axis can be obtained as follows

(20) $$\begin{array}{l} \alpha_{z}^{1.5.4}==\frac{\pi }{2}+\Delta \theta_{z4}-\Delta \theta_{z5}+\omega_{ie}T\sin L\\ \alpha_{z}^{1.4.3}=\frac{\pi }{2}+\Delta \theta_{z3}-\Delta \theta_{z4}+\omega_{ie}T\sin L\\ \alpha_{z}^{1.3.2}=\frac{\pi }{2}-\Delta \theta_{z3}+\omega_{ie}T\sin L\\ \alpha_{z}^{1.2.3}=-\frac{\pi }{2}+\Delta \theta_{z3}+\omega_{ie}T\sin L\\ \alpha_{z}^{1.3.4}=-\frac{\pi }{2}-\Delta \theta_{z3}+\Delta \theta_{z4}+\omega_{ie}T\sin L\\ \alpha_{z}^{1.4.5}=-(\frac{\pi }{2}+\Delta \theta_{z4}-\Delta \theta_{z5})+\omega_{ie}T\sin L\\ \alpha_{z}^{1.5.1}=-(\frac{\pi }{2}+\Delta \theta_{z5})+\omega_{ie}T\sin L \end{array}$$

By substituting into the gyro error equations of the strapdown inertial navigation system, we can obtain

(21) $$\left[\begin{array}{c} g_{z}^{1.5.4}\\ g_{z}^{1.4.3}\\ g_{z}^{1.3.2}\\ g_{z}^{1.2.5}\\ g_{z}^{1.2.3}\\ g_{z}^{1.3.4}\\ g_{z}^{1.4.5}\\ g_{z}^{1.5.1} \end{array}\right]=A\cdot \left[\begin{array}{c} (1+\Delta K_{gz})\cdot \frac{\pi }{2}\\ (1+\Delta K_{gz})\cdot \Delta \theta_{z3}\\ (1+\Delta K_{gz})\cdot \Delta \theta_{z4}\\ (1+\Delta K_{gz})\cdot \Delta \theta_{z5}\\ (1+\Delta K_{gz})\cdot (\omega_{ie}T\sin L)+B_{gz} \end{array}\right]$$

Through the 48 rotation procedures designed in this paper, the relevant error parameters, Δ*K_gx_*, Δ*K_gy_*, Δ*K_gz_*, *B_gx_*, *B_gy_*, *B_gz_*, Δ*θ_x_*_2_, Δ*θ_x_*_4_, Δ*θ_x_*_6_, Δ*θ_y_*_3_, Δ*θ_y_*_5_, Δ*θ_y_*_6_, Δ*θ_z_*_3_, Δ*θ_z_*_4_, Δ*θ_z_*_5_, can be identified.

By analyzing the outputs of the hemispherical resonator gyroscopes that are perpendicular to the *z*-axis during the 48 rotation procedures, the relevant errors,$\alpha_{z},\alpha_{y},\beta_{z},\beta_{x},\gamma_{y},\gamma_{x}$, of the three hemispherical resonator gyroscopes in the strapdown inertial navigation system can be identified.

By substituting the identified hexahedron errors into the accelerometer error model of the strapdown inertial navigation system, the relevant errors of the three accelerometers in the system can be identified.

## 5. Experimental Setup and Verifications

**Figure 5:**
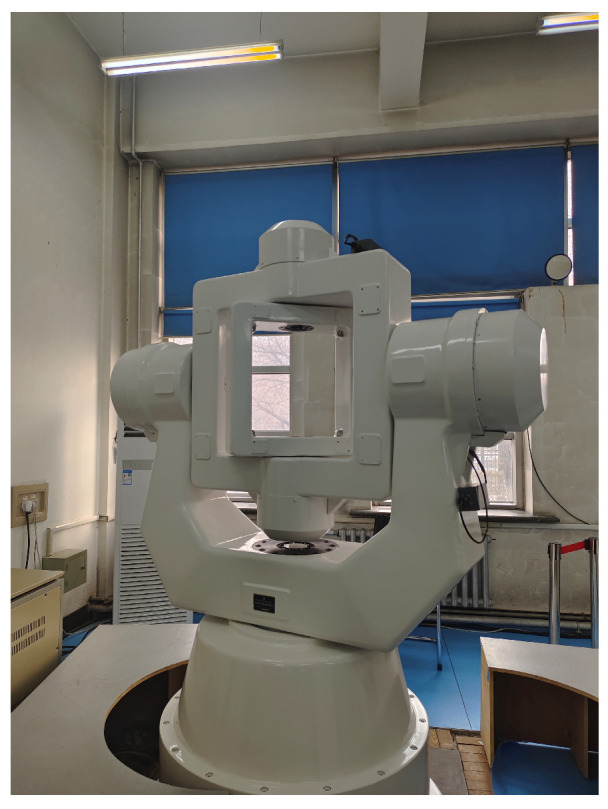
Experimental setup for validation.

**Figure 6:**
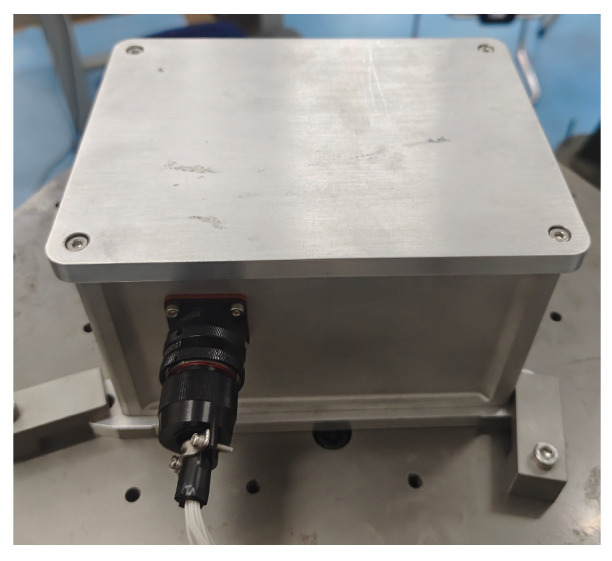
Physical diagram of HRG SINS.

To verify the correctness of the method proposed in this paper, a validation experiment was designed on a three-axis turntable. The turntable used in the validation experiment was mounted on a vibration-isolated foundation to suppress interference, and the ambient temperature during the experiment was maintained at 20 °C. The three-axis turntable is shown in Figure 5, the HRG SINS is shown in Figure 6. To rigorously validate the accuracy of the proposed method, a semi-physical simulation experiment was conducted using a high-precision three-axis turntable. The HRG’s measurement accuracy in SINS is higher than 0.01°/h, the accelerometer’s measurement accuracy is higher than 10^−4^ g. The three-axis turntable serves as the attitude reference standard. Its key technical specifications are Positioning Accuracy: 5″, Positioning Repeatability: 1″, and Rate Stability: Better than 0.001%.

Since measuring the geometric errors of a physical hexahedral fixture in situ involves significant uncertainty, this study utilized the turntable to simulate the fixture errors. By controlling the turntable to tilt the SINS by specific, preset angles during the static positioning phases, we created a ‘virtual’ hexahedron with known structural imperfections. These pre-set angular values provided by the turntable controller serve as the ‘Reference Truth’ for the structural errors. The SINS data collected under these simulated error conditions were processed using the proposed calibration algorithm. The identified error parameters were then compared against the Reference Truth.

The error model coefficients of the HRG SINS with hexahedron errors, identified using the traditional method are shown in Table 1. The hexahedron errors are simulated and applied to the HRG SINS by the three-axis turntable.

True value represents the error model coefficients of the HRG SINS calibrated using the discrete method on a three-axis turntable. Test values are the error model coefficients of the HRG SINS with hexahedron errors, identified using the traditional method. The error model coefficients of the HRG SINS with hexahedron errors, identified using the proposed method, are shown in Table 2.

The true values for the installation errors of the SINS accelerometers and gyroscopes were obtained through a standard high-precision discrete calibration on the turntable. The true values for the hexahedral fixture errors are the command values given to the three-axis turntable, which is the simulated error angles.

The experimental procedure involves simulating hexahedral errors via the turntable, collecting SINS data, and identifying the error model coefficients using both the proposed method and the traditional method. The identification results are then compared with the reference true values.

**Figure 7:**
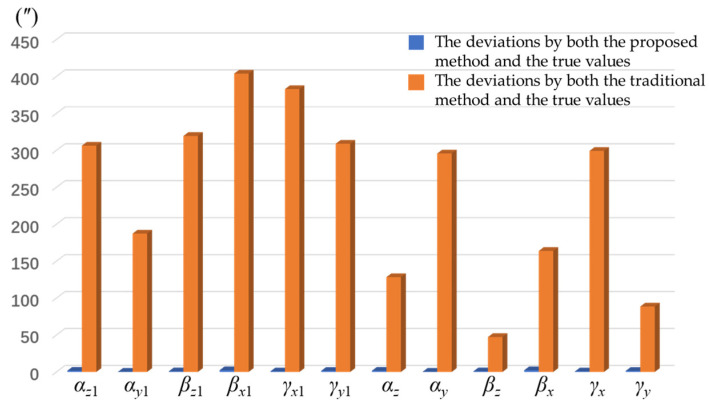
The deviations between the installation errors identified by both the traditional method and the proposed method and the true values.

Based on the analysis of Table 1 and Table 2, it can be concluded that the hexahedral structural errors of the HRG SINS significantly affect the identification of the installation errors for the tri-axial HRGs and accelerometers within the comprehensive error model. As shown in Figure 7, the deviations between the installation errors identified by both the traditional method and the proposed method and the true values are illustrated. Table 3 shows the comparison between traditional calibration methods and the proposed method.

The orange part shown in Figure 7 represents the deviations between the installation errors identified by both the proposed method and the true values. The blue part shown in Figure 7 represents the deviations between the installation errors identified by both the traditional method and the true values. By analyzing Figure 7, it can be concluded that the method proposed in this study effectively suppresses the impact of hexahedron errors on the identification of the HRG SINS error model coefficients.

However, certain limitations of the proposed method should be noted. First, the error modeling relies on the small-angle assumption for linearization; consequently, if the assembly errors or structural deformations are excessively large, the linear approximation may become insufficient. Second, while the method relaxes machining tolerance requirements, it still demands high positioning stability. Instances of positioning imprecision, such as loose contact between the hexahedron and the locating block, may still compromise the identification accuracy.

A key trade-off exists between hardware cost and operational complexity. This method significantly relaxes the mechanical precision requirements for the hexahedral fixture, reducing hardware costs. However, the proposed 24-position and 48-rotation scheme is more time-consuming than traditional 12-position schemes. Therefore, this method is best suited for applications where calibration accuracy is the priority and calibration time is a secondary constraint.

## 6. Conclusions

This paper presents a comprehensive on-field calibration method for strapdown inertial navigation systems based on hemispherical resonator gyroscopes, which reduced the influence of the errors of the hexahedral structural on the calibration accuracy of the SINS. A comprehensive error model for the on-field calibration of strapdown inertial navigation systems based on hemispherical resonator gyroscopes was established, and test plans were designed based on this error model. The proposed 24-position accelerometer calibration and 48-rotation gyro calibration schemes allow for simultaneous and accurate identification of sensor biases, scale factor errors, installation misalignments, and fixture-induced structural errors, providing a complete solution for high-precision SINS calibration.

Experimental validation on a high-precision three-axis turntable demonstrates that the proposed method significantly improves the calibration accuracy for both accelerometers and HRGs compared to conventional approaches, while maintaining robustness throughout different orientations and operating conditions. Furthermore, by calibration for the structural errors, the method reduces the mechanical precision requirements for the hexahedral fixture, lowering both the cost and complexity of on-field calibration. Overall, this study provides a robust and practical framework for high-precision calibration of HRG-based SINS and offers a methodology that can be extended to other advanced inertial navigation systems requiring accurate error modeling.

## Figures and Tables

**Table 1:** Experimental results using the traditional method.

Parameter	True Value	Test Value	Residual Error
Δ*K_ax_*(ppm)	347.826	341.435	−6.391
Δ*K_ay_*(ppm)	512.394	516.621	4.227
e*K_az_*(ppm)	589.173	597.088	7.915
*B_ax_*(μg)	431,645	433.962	2.317
*B_ay_*(μg)	624.435	615.151	−9.284
*B_az_*(μg)	748.815	752.321	3.506
*α_z_*_1_(″)	1342.7	1649.0	306.3
*α_y_*_1_(″)	987.4	1174.7	187.3
*β_z_*_1_(″)	421.6	102.2	−319.4
*β_x_*_1_(″)	725.9	1129.7	403.8
*γ_x_*_1_(″)	1113.2	1496.0	382.8
*γ_y_*_1_(″)	289.5	598.4	308.9
Δ*K_gx_*(ppm)	284.517	285.110	0.593
Δ*K_gy_*(ppm)	397.862	407.044	9.182
Δ*K_gz_*(ppm)	465.309	463.833	−1.476
*B_gx_*(°/h)	0.17248	0.17126	−0.00122
*B_gy_*(°/h)	0.19837	0.19895	0.00058
*B_gz_*(°/h)	0.25461	0.25274	−0.00187
*α_z_*(″)	1423.6	1552.0	128.4
*α_y_*(″)	879.4	583.7	−295.7
*β_z_*(″)	305.7	353.0	47.3
*β_x_*(″)	1167.2	1003.4	−163.8
*γ_x_*(″)	478.9	778.1	299.2
*γ_y_*(″)	1320.1	1231.5	−88.6

**Table 2:** Experimental results using the proposed method.

Parameter	True Value	Test Value	Residual Error
Δ*K_ax_*(ppm)	347.826	351.298	3.472
Δ*K_ay_*(ppm)	512.394	509.780	−2.614
Δ*K_az_*(ppm)	589.173	596.358	7.185
*B_ax_*(μg)	431,645	430.698	−0.947
*B_ay_*(μg)	624.435	629.466	5.031
*B_az_*(μg)	748.815	750.691	1.876
*α_z_*_1_(″)	1342.7	1340.4	−2.3
*α_y_*_1_(″)	987.4	988.1	0.7
*β_z_*_1_(″)	421.6	423.4	1.8
*β_x_*_1_(″)	725.9	725.4	−0.5
*γ_x_*_1_(″)	1113.2	1112.0	−1.2
*γ_y_*_1_(″)	289.5	290.6	1.1
Δ*K_gx_*(ppm)	284.517	282.201	−2.318
Δ*K_gy_*(ppm)	397.862	398.351	0.489
Δ*K_gz_*(ppm)	465.309	464.285	−1.024
*B_gx_*(°/h)	0.17248	0.17284	0.00036
*B_gy_*(°/h)	0.19837	0.19712	−0.00125
*B_gz_*(°/h)	0.25461	0.25545	0.00084
*α_z_*(″)	1423.6	1421.9	−1.7
*α_y_*(″)	879.4	879.7	0.3
*β_z_*(″)	305.7	306.6	0.9
*β_x_*(″)	1167.2	1164.8	−2.4
*γ_x_*(″)	478.9	479.7	0.8
*γ_y_*(″)	1320.1	1318.6	−1.5

**Table 3:** Comparison between traditional calibration methods and the proposed method.

Feature	Traditional Method	Proposed Method
Fixture Assumption	Assumes an ideal rigid hexahedron (perfect orthogonality and parallelism).	Accounts for structural errors (non-orthogonality and non-parallelism) of the hexahedron faces.
Mathematical Model	Angle-based rotation model; Fixture errors are ignored.	Vector-based model; Uses normal vectors to explicitly describe 9 structural error parameters.
Error Handling	Fixture errors are aliased into sensor biases, scale factors and installation errors, degrading accuracy.	Fixture errors are decoupled from sensor errors
Calibration Scheme	Typically 12-position (static) and 6-rotation (dynamic).	24-position (static) and 48-rotation (dynamic) to ensure observability of all parameters.
Hardware Requirement	High machining precision required for the hexahedron to minimize unmodeled errors.	Relaxed requirements; High calibration accuracy is achieved even with lower-precision fixtures.

## Data Availability

The raw data supporting the conclusions of this article will be made available by the authors on request.

## Abbreviations

**Symbol**	**Description**
*o_n_x_n_y_n_z_n_*	The navigation coordinate system
*o*_1_ *x*_1_ *y*_1_ *z*_1_	The slab coordinate system
*o_b_x_b_y_b_z_b_*	The hexahedron coordinate system
***n*** *_i_*	The outward normal vector of the face of the hexahedron
***s***	The normal vectors of the sensitive axes of the three accelerometers
***g***	The normal vectors of the sensitive axes of the three HRGs
*N^a^*	The output of the accelerometer
*N^g^*	The output of HRG
*α^i^.^j^*	The specific forces of accelerometers in the slab coordinate system
*α^i^.^j^.^k^*	The angular input of the HRG
Δ*K_ax_*,Δ*K_ay_*,Δ*K_az_*	The scale factor of the accelerometers in the SINS
*B_ax_*,*B_ay_*,*B_az_*	The bias of the accelerometers in the SINS
*α_y_*_1_,*α_z_*_1_,*β_x_*_1_ *β_z_*_1_,*γ_y_*_1_,*γ_x_*_1_	The installation errors of the accelerometers in the SINS
Δ*K_gx_*,Δ*K_gy_*,Δ*K_gz_*	The scale factor of the HRGs in the SINS
*B_gx_*,*B_gy_*,*B_gz_*	The bias of the HRGs in the SINS
*α_y_*,*α_z_*,*β_x_* *β_z_*,*γ_y_*,*γ_x_*	The installation errors of the HRGs in the SINS
